# Assessment of Fine Motor Abilities Among Children with Spinal Muscular Atrophy Treated with Nusinersen Using a New Touchscreen Application: A Pilot Study

**DOI:** 10.3390/children12101378

**Published:** 2025-10-12

**Authors:** Inbal Klemm, Alexandra Danial-Saad, Alexis R. Karlin, Rya Nassar-Yassien, Iuliana Eshel, Hagit Levine, Tamar Steinberg, Sharon Aharoni

**Affiliations:** 1 Department of Occupational Therapy, Schneider Children’s Medical Center of Israel, Petach Tikva 4920235, Israel; inbalke2@clalit.org.il (I.K.);; 2 Department of Occupational Therapy, Faculty of Social Welfare and Health Sciences, University of Haifa, Haifa 349002, Israel; asaad@univ.haifa.ac.il (A.D.-S.);; 3 Institute of Pediatric Neurology, Schneider Children’s Medical Center of Israel, Petach Tikva 4920235, Israel; 4 Institute of Pulmonology, Schneider Children’s Medical Center of Israel, Petach Tikva 4920235, Israel; 5 Gray Faculty of Medical & Health Sciences, Tel Aviv University, Tel Aviv 6139001, Israel

**Keywords:** spinal muscular atrophy, hand function, upper extremity motor function, Touchscreen Assessment Tool

## Abstract

**Background/Objectives:** Spinal Muscular Atrophy (SMA) is a genetic neurodegenerative disease characterized by severe muscle weakness and atrophy. Advances in disease-modifying therapies have dramatically changed the natural history of SMA and the outcome measures that are used to assess the clinical response to therapy. Standard assessment methods for SMA are limited in their ability to detect minor changes in fine motor abilities and in patients’ daily functions. The aim of this pilot study was to evaluate the feasibility and preliminary use of the Touchscreen-Assessment Tool (TATOO) alongside standardized tools to detect changes in upper extremity motor function among individuals with SMA receiving nusinersen therapy. **Methods:** Thirteen individuals with genetically-confirmed SMA, aged 6–23 years, eight with SMA type 2, and five with SMA type 3, participated. The patients continued the maintenance dosing of nusinersen during the study period. They were evaluated at the onset of the study, then twice more at intervals at least six months apart. Upper extremity functional assessments were performed via the TATOO and standardized tools: the Hand Grip Dynamometer (HGD), Pinch Dynamometer (PD), Revised Upper Limb Module (RULM), and Nine-Hole Peg Test (NHPT). **Results:** Significant changes in fine motor function were detected using the TATOO together with other standardized tools. Participants demonstrated notable improvements in hand grip strength and fine motor performance, as measured by the NHPT. The RULM results were not statistically significant for the total study group, particularly in ambulatory patients with SMA type 3. TATOO provided detailed metrics, and revealed enhancements in accuracy and speed across various tasks. However, given the small sample size, the lack of a control group, and the lack of baseline assessment before receiving therapy, these findings should be considered preliminary and exploratory. **Conclusions:** The findings suggest that the TATOO, alongside traditional assessment tools, offers a sensitive measure of fine motor function changes in patients with SMA. This study highlights the potential of touchscreen-based assessments to address gaps in current outcome measures and emphasizes the need for larger, multicenter studies that will include pre-treatment, baseline, and control data.

## 1. Introduction

Spinal muscular atrophy (SMA) is a rare autosomal recessive neuromuscular disease characterized by the progressive loss of motor neurons, and the subsequent atrophy of skeletal muscles and muscle weakness. The phenotypic spectrum is classically categorized into four subtypes according to the age of onset and the maximum motor milestone achieved. However, the classification has evolved to focus on milestone achievements (non-sitters, sitters, walkers) to better tailor interventions [1,2].

Prior to the development of gene- and RNA-based therapies, treatment for SMA largely consisted of supportive therapies including non-invasive ventilation and enteral feeding when the disease progressed. In recent years, disease-modifying therapies, including nusinersen, risdiplam, and onasemogene abeparvovec, have dramatically altered the course and outcomes of the disease. These advances have created a growing need for outcome measures that are sensitive to subtle motor changes and applicable across the SMA spectrum, in both adolescents and adults.

Clinical tools used to evaluate shifts in motor function in individuals with SMA focus on the physical examination of the range of motion of the musculoskeletal system and on related functional impairments. In addition, strength and timed tests are used to monitor aspects of function that reflect activities of daily living [1]. Assessment tools, such as the Revised Upper Limb Module (RULM) and Hand Grip Dynamometer (HGD) that were standardized before the emergence of current therapies, are still in use despite their limitations [3,4,5,6,7,8,9]. For example, the RULM is limited by a ceiling effect—meaning that, in patients with relatively preserved upper limb function, such as many with SMA type 3, changes in fine motor skills cannot be reliably detected [9,10]. Hand-held dynamometers provide quantitative measures of strength but do not reflect the complexity of fine motor skills [11]. The Nine-Hole Peg Test (NHPT) has been widely used as a standard tool for hand function assessment, including for various muscular atrophy conditions, whilst its revised version has been found to be more sensitive to upper extremity (UE) fatigue. Thus, it achieves better validation for assessing changes in dexterity in the SMA population [12,13]. Through the course of SMA treatment, individuals may undergo apparently minor changes in UE fine motor skills, which are undetected by traditional assessment methods, but may significantly impact daily functioning [10,14,15].

To address the gap in the functional assessment of fine motor skills for patients with neuromuscular disorders, a member of our team (A.D.S.) developed a Touchscreen Assessment Tool (TATOO) [16,17,18]. TATOO comprises multiple tasks, each designed to provide objective data on the performance of distinct functional components and motor skills necessary for touchscreen interaction [17]. Subsequent research has demonstrated the effectiveness of TATOO as an assessment tool for fine motor skills in diverse populations, including elderly individuals and children. Its applicability to individuals with type 2 diabetes mellitus is currently being investigated [15,18]. However, the tool has not been systematically studied among individuals with neuromuscular diseases like the SMA population.

The aim of this study was to evaluate the feasibility and preliminary use of TATOO alongside standard functional assessment tools to detect even subtle changes in UE motor function among individuals with SMA undergoing nusinersen therapy. We aimed to test three hypotheses. First, we hypothesized that the UE functional assessment via the TATOO application, along with standard assessment tools, including the HGD, the Pinch Dynamometer (PD), and the NHPT, would detect changes in functional motor skills. Second, we hypothesized that the RULM would not detect significant UE functional changes over the study period. Third, we hypothesized that correlations would be apparent between the standardized fine motor assessment tools (HGD, PD, and NHPT) and the TATOO assessment.

## 2. Methods

### 2.1. Study Design and Participants

This was a prospective pilot study conducted during the period 2020–2022 at the multidisciplinary neuromuscular clinic of Schneider Children’s Medical Center of Israel. As an exploratory study, no formal power calculation was performed. We aimed to assess the feasibility and to generate preliminary data for larger studies. The inclusion criteria were (1) genetically confirmed 5q SMA; (2) SMA type 2 or 3; (3) age ≥ 3 years; and (4) the ability to participate in standardized assessments. All participants received nusinersen therapy prior to study initiation and continued maintenance dosing every four months during the study period as part of standard care in our clinic. The patients were both ambulatory and non-ambulatory. The study population reflected the demographics of our clinical population, including individuals speaking Hebrew, Arabic, and Russian as their native tongues. Patients with comorbidities that could influence motor, sensory, and cognitive functions were excluded.

Written informed consent was obtained from all participants. The study was approved by the hospital review board (RMC-0031-20). All patients were evaluated during regularly scheduled clinic visits. Demographic and clinical data recorded at every visit included age, gender, SMA type, functional level, and dominant hand. Hand dominance was determined by asking participants about their preferred hand for daily tasks such as eating and writing, rather than by performing a standardized assessment. Each participant completed the standard assessments of UE gross and fine motor functions and the TATOO assessments of UE fine motor function, as detailed below. The participants underwent a total of three evaluation sessions: at the initiation of the study, at eight months (±2 months), and at 17 months (±5 months).

### 2.2. Assessment Tools

#### 2.2.1. Standard Assessment Tools for UE Function

The standard assessment tools included the HGD, PD, NHPT, and the RULM.

Two types of hand grip were assessed. Hand Grip Dynamometer (HGD) was assessed to measure hammer grip and lateral pinch strength using a calibrated JAMAR device, following the American Society of Hand Therapists protocol. These tasks were selected as they are reliable indicators of overall hand strength, and are feasible in individuals with SMA and variable abilities. Pinch Dynamometer (PD) was assessed to quantify precision pinch strength [3,4,5]. The NHPT was used to assess manual dexterity, defined as the ability to manipulate small objects via finger coordination in a timely manner. The test is scored by the number of seconds required to place nine pegs in a pegboard and then to remove them from the pegboard, first using the dominant then the non-dominant hand [6,7].

The RULM includes 19 items that correlate various proximal and distal UE functions. Each item is scored as 0 (unable), 1 (able, with modification), or 2 (able, no difficulty) [9,19]. All the assessments were conducted by an experienced occupational therapist and physical therapist.

#### 2.2.2. The Touchscreen Assessment Tool

TATOO provides an objective measure of the abilities required to use a touchscreen tool [16,17].

**Figure 1:**
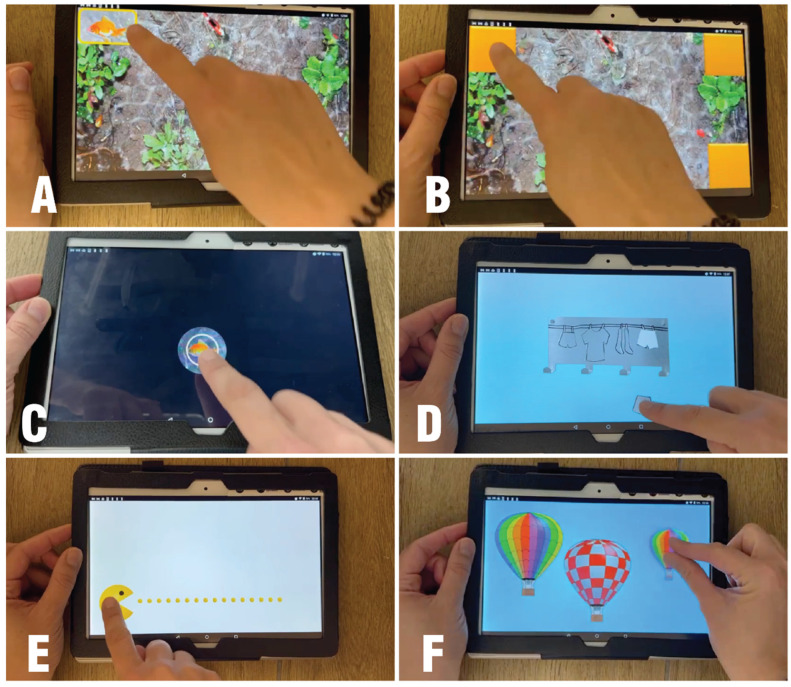
TATOO tasks. (**A**) touch and tap all corners; (**B**) double tapping; (**C**) tap on static and moving objects accurately; (**D**) drag objects in all directions; (**E**) drag objects along straight horizontal paths; and (**F**) pinching.

In the current study, the participants performed each of the seven following tasks: (1) touch and tap the entire screen area; (2) touch and tap all the corners; (3) double tapping; (4) tap on static and moving objects accurately; (5) drag objects in all directions; (6) drag objects along straight horizontal paths; and (7) pinching (Figure 1).

Performance on each task is summarized by numerical and graphical reports of the temporal metrics and accuracy metrics. The temporal metrics (in seconds) include reaction time, task duration, touch time (the total time in seconds during which the finger touches the screen surface), and flight time (the time in seconds during which the finger does not touch the screen surface). The accuracy metrics include the number of taps, drag attempts, drag completed successfully, and touch outside (the number of errors) [18]. As TATOO is a novel tool in SMA, this study focused on feasibility and sensitivity, and not on reliability and construct validity, which should be investigated in future studies.

### 2.3. Statistical Analysis

The data were analyzed using SPSS^®^ version 27. Descriptive statistics were used to summarize demographic and clinical data. The normality of data distribution was assessed using the Shapiro–Wilk test. As most variables did not meet the assumption of normality, and due to the small sample size (*n* = 13), we employed non-parametric tests throughout the analysis.

First, we evaluated the differences in the performance of the participants’ right and left hands during the first assessment to scrutinize whether hand dominance influences performance. Second, we assessed the changes between the first and last assessments for each outcome measure. Due to the exploratory nature of this pilot study, multiple comparisons were not corrected. Instead, emphasis was placed on the effect size, which was calculated as r = Z/√N to quantify the magnitude of observed changes. Interpretations were based on Cohen’s criteria (small: r = 0.1–0.3, moderate: r = 0.3–0.5, large: r > 0.5). We report both statistically significant findings (*p* ≤ 0.05) and statistical trends (0.05 < *p* < 0.10). These are accompanied by moderate-to-large effect sizes, which may indicate clinically meaningful changes warranting investigation in further studies. Spearman’s rank correlation coefficient test was used to examine the correlation between the parameters of standardized fine motor tools (HGD, PD, NHPT), assessed separately for each hand.

## 3. Results

A total of 16 individuals with SMA were recruited. After excluding three individuals due to missed evaluations, the participants included 13 individuals aged 6–23 years. Eight were diagnosed with SMA type 2 and five were diagnosed with SMA type 3. Their clinical and demographic characteristics are shown in Table 1.

### 3.1. Comparison of Right- and Left-Hand Performance

At the first assessment, no significant differences were found between right- and left-hand performance across all the standard assessment tools. Based on this finding, subsequent statistical analyses were conducted without stratification by hand dominance but only for right–left performance disparities.

### 3.2. Comparison Between First and Last Assessments—Standard Tools

Longitudinal analysis revealed significant improvements in several standardized measures of upper limb function. HGD increased significantly in both hands (z = −2.76, *p* < 0.01).

Manual dexterity, as measured by the NHPT, showed asymmetric improvement patterns. The left hand demonstrated statistically significant improvement with a reduced task completion time (median change from 28.35 to 23.03 s; Z = −2.22, *p* = 0.026, r = 0.67). The right hand paradoxically showed a slight increase in completion time, though this change was not statistically significant (Z = −1.69, *p* = 0.09, r = 0.51). Despite the lack of statistical significance, the large effect size suggests potential clinical relevance requiring further investigation.

Pinch strength measurements assessed by PD revealed minimal changes, with only the right hand showing a trend toward improvement (Z = −1.34, *p* = 0.18, r = 0.37). This represents a moderate effect size without reaching statistical significance (See Table 2).

### 3.3. Comparison Between First and Last Assessments Using TATOO

Using the TATOO application, significant improvements were observed primarily in right-hand performance for both temporal metrics and accuracy metrics across the various tasks. The temporal metrics improved markedly. The median reaction time decreased by 19% (from 3.14 to 2.54 s, z = −2.97, *p* < 0.01). The median flight time decreased by 18% (from 9.49 to 7.79 s, z = −3.11, *p* < 0.01). The median test duration decreased by 20% (from 14.24 to 11.44 s, z = −2.04, *p* < 0.01). The accuracy metrics also improved. The median number of errors of touch outside the desired area decreased by 78% (from 1.29 to 0.29, z = −1.43, *p* < 0.05). The total drag attempts decreased from 10 to 9 (z = −2.02, *p* < 0.05). Metrics such as touch time, the number of taps, and the number of drags completed successfully demonstrated moderate effect sizes despite not reaching statistical significance (Table 3). This suggests potential clinical relevance in a larger sample.

In contrast to the above, fewer significant improvements were observed with left hand performance. A significant reduction by 10% was observed only in reaction time, from 2.77 to 2.47 s (z = −2.27, *p* < 0.05). This asymmetric pattern may reflect differential use in daily activities or in hand-specific therapeutic responses. Other left-hand metrics—including flight time, task duration, and accuracy-related variables, demonstrated small to moderate effect sizes without statistical significance. This highlights consistent trends but requires confirmation in future studies (see Supplemental Table S1).

### 3.4. Comparison Between First and Last Assessments—RULM

The first and last RULM assessments showed contrasting patterns between the SMA total group and the SMA subtypes. Considering all the participants analyzed, the RULM results revealed no statistically significant differences (z = −1.44, *p* = 0.15) although a moderate effect size (r = 0.4) showed a trend of change. The first median score was 26 (interquartile range [IQR]: 13.5–36.5) compared with 24 (IQR: 14–37) at the last assessment. Subgroup analysis revealed important distinctions. Participants with SMA Type 2 demonstrated a statistically significant improvement, with median scores increasing from 16 to 18 points (Z = −2.39, *p* = 0.02), representing an 11% gain in upper limb function. This improvement is clinically meaningful in this population in which first-time function is limited.

In contrast, participants with SMA Type 3 showed no measurable change (first time median 36.5/37, final 37/37; Z = −1.07, *p* = 0.29).

These findings underscore the RULM’s variable utility, namely its appropriateness for monitoring Type 2 patients with moderate impairment, and inadequacy for Type 3 patients who approached maximum scores.

### 3.5. Correlations Between Standardized Tools and TATOO

Finally, we examined the correlations between measurements of standardized fine motor tools and TATOO for both hands. In the right hand, the parameter “number of taps” of the first task (“Touch all screen areas”) correlated significantly with HGD (r = 0.81, *p* = 0.01) and PD (r = 0.74, *p* = 0.02). This suggests that tap frequency reflects the underlying force generation capacity. As expected, tap frequency correlated negatively with NHPT (r = −0.63, *p* = 0.03), indicating that faster tapping corresponds to better manual dexterity.

For both hands, the “pinch ability” task duration showed strong correlations with NHPT performance. For the right hand, rs = 0.67, *p* = 0.02. For the left hand, rs = 0.90, *p* < 0.001. This particularly strong left-hand correlation suggests that TATOO’s pinch task captures similar fine motor control demands as traditional pegboard tasks.

Taken together, these findings provide the preliminary evidence of convergent validity for TATOO, as the temporal and accuracy-based measures demonstrate consistent associations with the established fine motor performance tools. A comprehensive overview of all correlation analyses is provided in Supplemental Table S1.

## 4. Discussion

To date, only a few studies have evaluated innovative tools to assess fine motor skills or dexterity in the SMA population, particularly in individuals undergoing treatment [14]. This raises the need to establish standardized fine motor assessment tools that are sensitive to subtle changes in function, accessible to all individuals with SMA regardless of functional status or SMA type, and efficient and easy to administer. Our study demonstrated that TATOO, alongside standardized fine and gross motor assessment tools, was able to detect significant changes in motor function among individuals with SMA types 2 and 3 receiving nusinersen over a 12–18-month period.

Importantly, TATOO provided detailed temporal and accuracy-based metrics, including reaction time, task duration, and the accuracy of finger movements. These parameters are directly relevant to daily activities that rely on touchscreen technology, underscoring the ecological validity of this tool [16,17,18]. Taken together, our preliminary findings suggest that TATOO may serve as a complementary, sensitive assessment method for monitoring functional changes in the SMA population.

In contrast to the above, the RULM alone was insufficient to capture significant changes at the group level, which was consistent with prior studies [20,21]. Literature reviews and meta-analyses in recent years have indicated that the RULM is hampered by a ceiling effect. Accordingly, the assessment is more effective for detecting motor changes among SMA Type 2 and non-ambulatory Individuals; and less for individuals with SMA Type 3, ambulatory, and with higher baseline functional abilities [22,23,24,25]. The median score of our participants with SMA type 3 was 36.5/37 at the first test, which may explain the lack of significant findings. Compared to our other findings, our study suggests that the ceiling effect may be mitigated by a combination of assessments with TATOO, HGD and PD, and the NHPT.

We report significant improvements over time in the hand grip strength of both hands, as measured by the HGD. This is consistent with studies that found statistically significant improvements in hand grip strength among young and adult participants with SMA types 2, 3, and 4, compared to assessments before receiving therapy [10,20,21,26]. However, in our study, the participants had already initiated nusinersen therapy at the time of the first assessment, which may partly explain the differences with prior studies.

Regarding fine motor skills and dexterity, the results revealed significant improvements in performance time for the left hand, and a trend towards improvement for the right hand using the NHPT. These findings align with the study by Gu, Minsu, and Hyun-Ho Kong, published in 2021 [27]. They also reported significant improvements in dexterity for both hands among five participants with SMA type 2, following 18 months of novel therapy using a similar standard dexterity test, the PERDEU Pegboard. This improvement may be attributed to the practice learning effect, which leads to relatively permanent changes in the capability for skilled performance, and which is considered the most crucial factor for improvement in performing motor skills [28].

Finally, our study revealed a strong significant correlation between one of our parameters (“number of taps”) in TATOO and all the standardized motor assessment tools. This provides evidence of convergent validity, indicating that TATOO captures dimensions of motor function comparable to established measures of speed, accuracy, stability, strength, precision, durability, and range of motion. In addition, the tool’s other metrics offer further functional and pragmatic assessments. We also found a significant correlation between the NHPT (measured by performance time) and the test duration parameter of the Pinch Ability task in TATOO. In both tests, the participants are required to “pinch objects” as fast as they can in all areas of the screen or peg board. Therefore, the resemblance between these tasks likely explains the positive correlation. Other TATOO metrics inconsistently correlated with standardized tools, highlighting that the application may assess both overlapping and unique facets of fine motor performance. Gabyzon, M. et al. [15] examined correlations of TATOO with tests of hand strength and manual dexterity among independent community-living older individuals. They reported no significant correlations between most of the TATOO-measured parameters and the parameters of those assessment tools. However, they did note a moderately positive trend between the number of double taps and manual dexterity (r = 0.32, *p* = 0.07). Taken together, these diverse findings underscore the complexity of fine motor skill assessment and highlight the potential of TATOO to provide nuanced insights across various populations and conditions.

In the future, measurement tools like TATOO may prove to be a welcome alternative to standardized assessment tools. This is due both to their accurate and precise measuring of functional fine motor ability, and their distinct advantages over prior tools. Indeed, our study has revealed many potential advantages of this tool. A crucial advantage of TATOO is its ability to measure functional changes in individuals with SMA, regardless of age and functional limitations, including those limited by contractures, thus effectively eliminating both floor and ceiling effects. Furthermore, TATOO does not need to be modified or adapted according to functional ability, making the application a more accessible and standardized tool than the NHPT or HGD. Moreover, TATOO does not require the use of specialized equipment. Indeed, the test administrator only needs an electronic tablet with the application downloaded.

Finally, the now nearly universal use of touchscreen tools to perform daily tasks of living, including academic pursuits, social engagement and communication, and purchase of food and other essentials provides a clear opportunity for an innovative approach to ecological clinical assessments. During the present period, the primary advantage of TATOO is its ability to simulate nearly universal tasks of daily living, thereby providing meaningful and pragmatic functional evaluations.

The study had several limitations, which limit the real-world usability of TATOO as a stand-alone tool, yet provide opportunities for further investigation. Firstly, we were unable to test TATOO with a demographically similar control population. Though validation is in process, we do not currently have the norms for a healthy population nor the norms for individuals with SMA along various functional stratifications. This has limited our ability to provide standardized “scores” to summarize an individual’s fine motor abilities. Rather, we measured motor abilities across an abundance of parameters. As our current quantification technique may well prove burdensome to providers in the future, it is critical to devise a simple scoring metric based on standardized measurements. Secondly, we recruited participants when they were already receiving nusinersen therapy. Therefore, we did not attain a pre-treatment assessment, and the treatment course itself varied among participants. Nevertheless, we did demonstrate the usability of the tool in conjunction with other tools to measure changes during treatment. Furthermore, given timeframe discrepancies between assessments, we were unable to compare baseline standardized assessments to the TATOO assessment. In addition, the time interval between assessments was not consistent among all participants due to scheduling disruptions related to the COVID pandemic at the time. This mostly impacted the timing of the second visit. Indeed, we also found that the time interval between first and second assessments was too short for our findings to be significant for most patients. For these two reasons, we used only the first and last assessments in the final analysis. Finally, our small sample size limited our statistical analysis, and we cannot exclude a potential learning effect with repeated exposure to TATOO tasks. No formal learning-effect analysis was conducted. Further studies may opt to split groups according to SMA type or functional status.

## 5. Conclusions

The results of this pilot study suggest that the skills necessary for the operation of touchscreen devices entail specific capabilities that are generally not captured by traditional assessment tools. The clinical implication is that the hand function assessment toolbox should be expanded. Tools such as TATOO can be used to capture the skills required for touchscreen manipulation in the context of the modern digital milieu. We hope that this pilot study may influence the development of new tools aimed to better detect subtle functional changes in individuals with SMA, particularly those receiving novel therapies. This may pave the way to tailor therapies and even contribute to the development of personalized functional technologies using similar touchscreen tools. Finally, the use of such tools may contribute to the developing knowledge about the influence of these gene- and RNA-based therapies on the motor functions of the upper extremity in the SMA population.

## Figures and Tables

**Table 1:** Clinical and demographic characteristics of the patients, *n* = 13.

Variable	Total Patients
**Age, years**	
Mean (SD)	14.1 (±5.81)
Median (p25–p75)	15 (7.9–19.15)
**Gender, *n* (%)**	
Male	6 (46.2)
Female	7 (53.8)
**SMA type, *n* (%)**	
Type 2	8 (61.5)
Type 3	5 (38.5)
**SMN2 copy number, *n***	
3	10
4	3
**Dominant hand, *n* (%)**	
Right	11 (84.6)
Left	2 (15.4)
**Ambulatory, *n* (%)**	
No	8 (61.5)
Yes	5 (38.5)

SMA: spinal muscular atrophy.

**Table 2:** Comparison between participants’ first and last assessments using standard tools.

Variable	Hand	First Assessment	Last Assessment	Z	Effect Size	Relative Size
		Mdn.	Mdn.			
HGD (kg)	R	4.65	6.05	−2.76 **	0.80	
	L	4.50	5.75	−2.76 **	0.80	
PD (kg)	R	1.7	1.9	−1.34 ^+^	0.37	Moderate
	L	1.6	1.1	−0.42	0.12	
NHPT (sec.)	R	22.90	23.32	−1.69 ^+^	−0.51	Large
	L	28.35	23.03	−2.22 *	0.67	

* *p* < 0.05, ** *p* < 0.01, ^+^ Clinical change without statistical significance. The relative effect size was added: small (r = 0.1–0.3), moderate (r = 0.3–0.5), large (r > 0.5). Mdn. = median score. HGD = Hand grip Dynamometer, PD = Pinch Dynamometer, NHPT = Nine-Hole Peg Test, R = Right Hand, L = Left Hand. Kg = kilograms, sec. = seconds.

**Table 3:** Comparison between participants’ first and last assessments using the TATOO application.

Variable	Hand	First Assessment	Last Assessment	Z	Effect Size	Relative Size
		Mdn (IQR)	Mdn (IQR)			
Reaction time (sec.)	R	3.14 (1.14)	2.54 (0.73)	−2.97 **	0.82	
Flight time (sec.)	R	9.49 (2.01)	7.79 (2.18)	−3.11 **	0.86	
Touch time (sec.)	R	4.14 (1.68)	4.00 (1.23)	−1.22 +	0.33	Moderate
Test duration (sec.)	R	14.24 (4.23)	11.44 (3.32)	−2.04 **	0.84	
Number taps (*n*)	R	138 (63.50)	132 (63.50)	−1.26 +	0.35	Moderate
Touch outside (*n*)	R	1.29 (2.36)	0.29 (0.64)	−1.43 *	0.67	
Drag completed successfully (*n*) #	R	8 (0)	8 (0)	−1.41 +	0.39	Moderate
Total drag attempts (*n*)	R	10 (2.50)	9 (0.50)	−2.02 *	0.56	

* *p* < 0.05, ** *p* < 0.01, + Clinical change without statistical significance. Relative effect size was added. Mdn = median score, IQR = interquartile range. R = right, L = left, sec. = seconds, *n* = number. # The median score range in the first assessment was 1 (7–8), and in the last assessment was 0 (8–8).

## Data Availability

The research data are not shared.

## Supplementary Materials

The following supporting information can be downloaded at: https://www.mdpi.com/article/10.3390/children12101378/s1.

## Abbreviations

UE:	Upper Extremity
HGD:	Hand Grip Dynamometer
NHPT:	Nine-Hole Peg Test
PD:	Pinch Dynamometer
RULM:	Revised Upper Limb Module
SMA:	Spinal Muscular Atrophy
TATOO:	Touchscreen-Assessment Tool
