# Supplementary material for: Assessment of Fine Motor Abilities Among Children with Spinal Muscular Atrophy Treated with Nusinersen Using a New Touchscreen Application: A Pilot Study
Source: Children (Basel). 2025 Oct 12;12(10):1378. doi: 10.3390/children12101378 (PMC12562439; doi:10.3390/children12101378)
Supplement: Supplementary file 1 [file children-12-01378-s001.zip › children-3823218-supplementary.pdf]

Supplemental Material Table S1

Variable	Hand	First Assessment	Last Assessment	Z	Effect Size	Relative size
Reaction Time (sec.)	L	2.77 (.89)	2.47 (.61)	-2.271*	.63	
Flight Time (sec.)	L	7.90 (2.90)	7.33 (1.34)	-1.01	.28	Small
Touch Time (sec.)	L	3.64 (2.55)	4.05 (1.13)	-.59	.16	Small
Test Duration (sec.)	L	12.67 (3.30)	12.23 (3.14)	-1.36	.38	Moderate
Number Taps (n)	L	116 (61)	114.50 (38.75)	-.05	.01	Small
Touch Outside (n)	L	.43 (.93)	.57 (.36)	-.58	.16	Small
Drag Completed Successfully (n)	L	8 (0)	8 (0)	-.89	.24	Small
Total Drag Attempts (n)	L	9 (1.5)	9 (1)	-.94	.26	Small
